# CXCR7 stimulates MAPK signaling to regulate hepatocellular carcinoma progression

**DOI:** 10.1038/cddis.2014.392

**Published:** 2014-10-23

**Authors:** L Lin, M-M Han, F Wang, L-L Xu, H-X Yu, P-Y Yang

**Affiliations:** 1 Proteomics and System Biology Lab, Institutes of Biomedical Sciences of Shanghai Medical School, Fudan University, Shanghai, China; 2 State Key Laboratory of Genetic Engineering, School of Life Sciences, Fudan University, Shanghai, China; 3 Department of Chemistry, Fudan University, Shanghai, China

## Abstract

The CXCL12/CXCR4 axis has been posited widely to have significant roles in many primary tumors and metastases. It is known that CXCR7 can also be engaged by CXCL12, but the exact function of CXCR7 is controversial. This prompted us to investigate the expression, specific function and signal transduction of CXCR7 in hepatocellular carcinoma (HCC). In this study, CXCR7 and CXCR4 were differentially expressed in nine cell lines of HCC, and that elevated expression of both CXCR7 and CXCL4 were correlated with highly metastatic ability of HCC cells. Moreover, CXCR7 expression was significantly upregulated in metastatic HCC samples compared with the non-metastatic ones by staining of high-density tissue microarrays constructed from a cohort of 48 human HCC specimens. CXCR7 overexpression enhanced cell growth and invasiveness *in vitro*, and tumorigenicity and lung metastasis *in vivo*. By contrast, CXCR7 stable knockdown markedly reduced these malignant behaviors. In addition, it was observed that alterations in CXCR7 expression were positively correlated with the phosphorylation levels of mitogen-activated protein kinase (MAPK) pathway proteins. Targeting extracellular regulated kinase pathway by using U0126 inhibitor or using CCX771, a selective CXCR7 antagonist, drastically reduced CXCR7-mediated cell proliferation. Importantly, by using human biotin-based antibody arrays, several differentially expressed proteins were identified in CXCR7-overexpression and depletion groups. Comparative analysis indicated that upstream regulators including TP53 and IL-6 were involved in CXCR7 signal transduction. CXCR7 expression was further proved to regulate expression of vascular endothelial growth factor A and galectin-3, which may contribute to tumor angiogenesis and invasiveness. Consequently, elevated expression of CXCR7 contributes to HCC growth and invasiveness via activation of MAPK and angiogenesis signaling pathways. Targeting CXCR7 may prevent metastasis and provide a potential therapeutic strategy for HCC.

Liver cancer is the third most common cause of cancer-related mortality worldwide.^1^ Although surgical and medical interventions becomes available, over 70% patients with metastasis or recurrence have little chance of survival.^2^ High rate of tumor metastasis to lung after hepatectomy represents a highly organized and organ-selective process. Emerging evidences indicate that several chemokines and their receptors appear to be directly implicated in mediating the biological cascade of sequential events, leading to tumor formation and metastasis.^3,4^

Chemokines are small secreted proteins (8–12 kDa) that share structural similarities, and members of this molecular superfamily can be subdivided into four classes, the C-C, C-X-C, C and C-X3-C chemokines, depending on the location of the first two conserved N-terminal cysteine residues that built disulfide bonds to two other cysteine residues within the protein sequence.^5^ To date, about 50 chemokines and ~25 chemokine receptors have been identified, thus some ligands exclusively bind to one receptor, others may interact with several receptors.^6^ Apart from their initially discovered and best-known function in trafficking of leukocytes,^7^ these soluble chemokines also influence organ development and physiological homeostasis via their respective G-protein-coupled receptors (GPCRs).^8^

Mounting studies indicate the involvement of chemokines and their respective receptors in tumor biology during the last decade. Malignant cells can express chemokine receptors and respond to chemokine gradients and this may be related to the growth and spread of cancer.^9^ In particular, CXCL12, which is also called stromal-derived factor-1, and its receptor CXCR4 have attracted tremendous interest, owing to their critical role in determining the metastatic destination of breast cancer cells.^10^ Shortly thereafter, researches revealed that CXCR4 is one of the most common chemokine receptors that cells from at least 23 different types of cancer express this receptor and respond to its ligand CXCL12.^11, 12, 13^ Since CXCL12/CXCR4 axis has a vital role in embryonic development of the hematopoietic, cardiovascular and nervous systems, the deficiency of this axis leads to perinatal lethality.^14, 15, 16^ This raises questions concerning the feasibility of CXCR4 as the therapeutic target in a range of cancers.

For a long time, CXCR4 was thought to be the only receptor for CXCL12, however, recent studies reported that CXCL12 also binds to CXCR7 with 10 times higher affinity than to CXCR4.^17,18^ CXCR7 is expressed in zebrafish and frog and is highly conserved in mammals.^19^ In the immune system, intracellular expression of CXCR7 has been found in T cells and monocytes,^20^ and its surface expression on B cells, plasmacytoid DCs, NK cells and basophils.^21^ In addition, very small but functional level of CXCR7 is observed on the surface of T cells, indicating that the intracellular pool of CXCR7 recycles back to the cell membrane and becomes activated.^17,22^ CXCR7 plays its important exclusive role in the proper development of the heart, particularly cardiac valves and its expression pattern in brain cells is also documented.^23,24^ It is known that CXCR7 is expressed on activated endothelial cells, fetal liver cells and on tumor-associated blood vessels as well as distinct tumor cells, but not on most nontransformed cells.^17,25^ Growing evidence has suggested that membrane-associated CXCR7 expression is elevated on many malignant cells, including breast,^18,25^ lung,^25^ prostate,^26^ pancreatic^27^ and glial cancer cells.^28^ Based on current literature, in glioma, CXCR7 is a functional receptor for CXCL12 and mediates anti-apoptotic/survival effects;^28^ whereas in breast cancer, CXCL12 enhanced CXCR7-mediated tumorigenicity and invasiveness by activating proinflammatory STAT3 signaling.^29^ Moreover, CXCR7 may also has CXCR4-independent functions as it provides growth and survival advantages even without ligand binding.^18,30,31^ How CXCR7 mediates the multiple functions has remained a mystery. Unlike most other CXC chemokine receptors, CXCR7 lacks the specific DRYLAIV motif, hence CXCR7 fails to mobilize Ca^2+^ from intracellular stores or extracellular sources after ligand engagement, which is the hallmark of chemokine receptor activation.^18^ Thus our interest is to explore the role and the mechanism of CXCR7 in hepatocellular carcinoma (HCC) progression.

## Results

### Upregulation of CXCR7 is associated with metastasis of HCC

**Figure 1:**
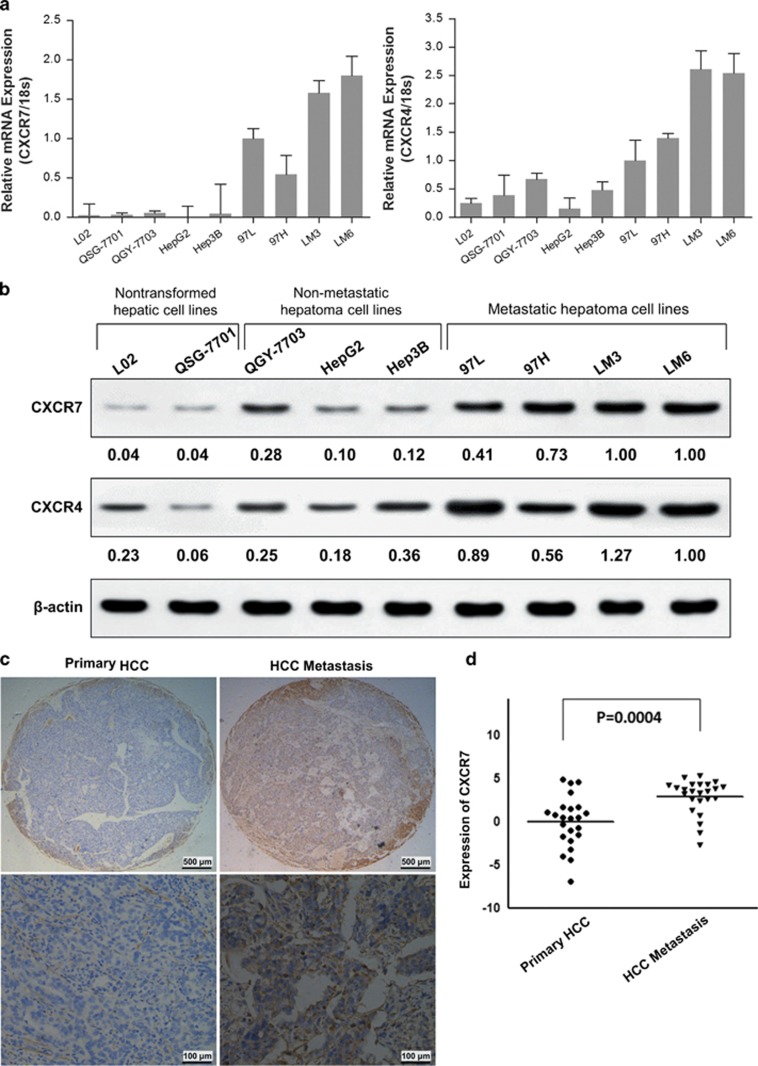
The expression levels of CXCR7 and CXCR4 in HCC cell lines and clinical specimens. (**a**) Expression levels of CXCR7 and CXCR4 were determined in a panel of established human hepatoma cell lines by qRT-PCR and normalized against an endogenous control (18S RNA). (**b**) CXCR7 and CXCR4 proteins were rarely expressed in the nontransformed hepatic cell lines L02 and QSG-7701, moderately expressed in non-metastatic HCC cells, but was dramatically overexpressed in highly metastatic cells (97L, 97H, LM3 and LM6) by western blot analysis. Semi-quantitative assessments were conducted using Quantity One software and quantitative data was also shown in **b**, normalized against loading control (*β*-actin). (**c**) Representative elements of HCC tissue microarray demonstrated weak staining in primary HCC (left panel) but strong staining in HCC with metastasis (right panel). Original magnification, × 40; scale bars, 500 *μ*m. Micrographs were taken at an original magnification × 200 in the lower panel, where the black bars represent 100 *μ*m. (**d**) Quantitative histologic evaluation of CXCR7 was analyzed by Image-Pro Plus 6.0 (*P*<0.001)

Expression of CXCR7 and CXCR4 were first examined in a panel of human HCC cell lines with varying metastatic capability. Quantitative reverse-transcription PCR (qRT-PCR) analysis showed that CXCR7 mRNA expression level was significantly increased in seven established HCC cell lines relative to the nontransformed hepatic cell lines L02 and QSG-7701. Moreover, highly metastatic cells (97L, 97H, LM3 and LM6) expressed much higher levels of CXCR7 than those low metastatic ones, including QGY-7703, HepG2 and Hep3B (Figure 1a). The western blotting experiment confirmed the results of qRT-PCR (Figure 1b), indicating the involvement of CXCR7 in HCC aggressiveness. In addition, CXCR4 mRNA and protein expressions were detected in all HCC cell lines. Lowly aggressive cell lines had small amounts of CXCR4, whereas reverse effects were observed in highly aggressive ones (Figures 1a and b). Of interest, CXCL12 mRNA expression was undetectable in most of the HCC cell lines but only in 97H and LM3 cell lines (data not shown), indicating the involvement of CXCL12/CXCR7/CXCR4 axis in HCCs.

To further evaluate the association of CXCR7 with HCC metastasis, a high density tissue microarray was constructed from clinical samples that included 24 primary HCCs without metastasis and 24 HCCs with metastasis. Representative images presented that membranous locations of CXCR7 were observed in most cases, and in some cases also expressed in cytoplasma but rarely expressed in nuclear (Figure 1c). Quantitative immunohistochemistry evaluation showed that metastatic HCC specimens have obviously higher CXCR7 level than the localized ones (*P*=0.0004, *t*-test; Figure 1d). These results suggest that high expression of CXCR7 correlates positively with HCC metastasis.

### CXCR7 promotes cell proliferation and induces G_0_/G_1_ to S phase transition in HCCs

**Figure 2:**
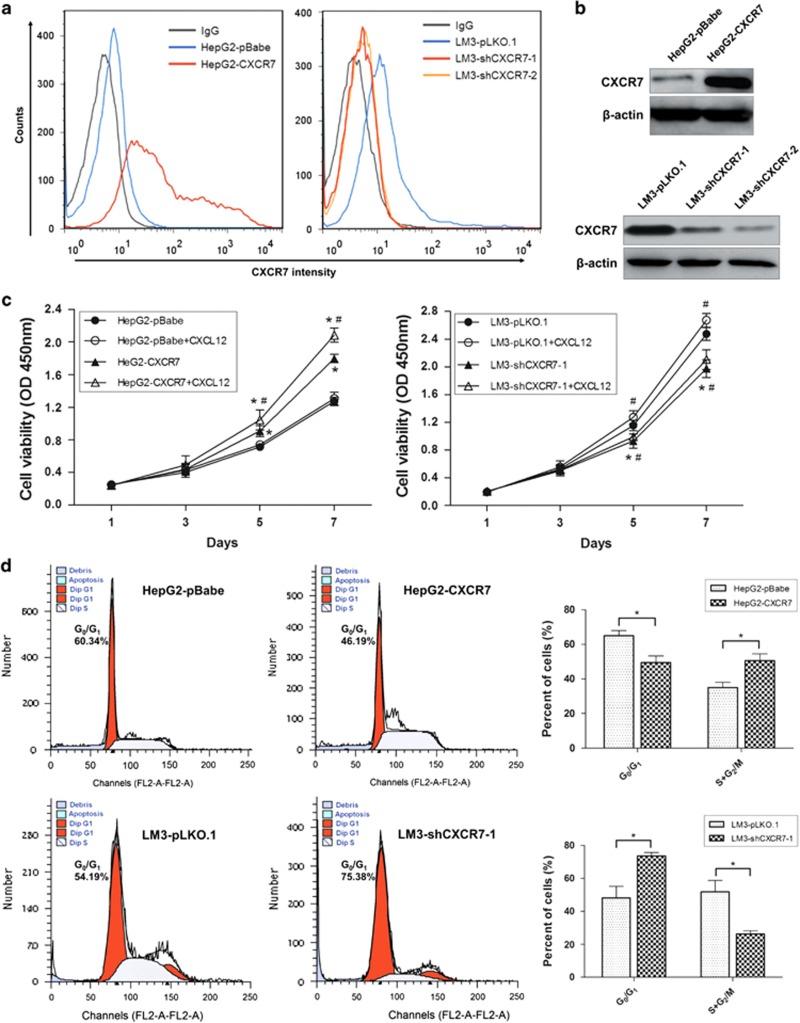
CXCR7 modulates phenotype of HCC cells *in vitro*. (**a**) Exogenous CXCR7 was expressed in HepG2 cells transfected with the pBabe vector. Both shCXCR7-1 and shCXCR7-2 were used to knockdown CXCR7 in LM3 cells. Parental cells with empty vector (pBabe or pLKO.1) were used as control. The surface expression of CXCR7 were evaluated by FACS analysis, using a phycoerythrin (PE)–anti-CXCR7 monoclonal antibody to detect CXCR7 expression, and a matched PE mouse IgG served as isotype control. (**b**) Western blot analysis comfirmed the expression of CXCR7 in HepG2- and LM3-transfected cells. (**c**) Cell proliferations were examined by CCK-8 experiments for HCC cell lines in response to altered CXCR7 levels with or without CXCL12 stimulation. * denotes significant difference from respective controls (as compared with cells transfected with empty vector); ^#^, significant from nontreatment controls by CXCL12 (100 ng/ml) stimulation (*P*<0.05) for mean±S.D. of five samples per condition. Error bars represent ±S.D. (**d**) Cell cycle profile was analyzed by FACS, upregulation of CXCR7 in HepG2 cells trigered cell cycle progression from G_0_/G_1_ to S+G_2_/M phase, while depletion of CXCR7 in LM3 cells altered the cell cycle by arresting the G_0_/G_1_ to S phase transition. The summary graphs were presented for the cell cycle assay that was outlined in **c**. Data represent the mean±S.D. of three independent experiments (*P<0.05)

To characterize the role of CXCR7 in HCC, we stably overexpressed CXCR7 in HepG2 and Hep3B cells, which have no metastatic potential and low endogenous CXCR7 expression levels, meanwhile, depleted CXCR7 in LM3 and 97H, which have high metastatic potential and high endogenous CXCR7 expression levels. Gene overexpression and silencing were monitored by fluorescence-activated cell sorting (FACS) and western blotting (Figures 2a and b, Supplementary Figure S1). CCK-8 experiments showed that cells overexpressing CXCR7 proliferated more rapidly than the control cells, whereas cells with depleted CXCR7 grew more slowly than control (Figure 2c, Supplementary Figures S1 and S2). Moreover, the CXCR7-transfected HepG2 cells showed a substantial increase in cell numbers in the presence of CXCL12 (100 ng/ml), compared with nontreatment ones; but proliferation was not increased in HepG2-pBabe cells by CXCL12 stimulation. Strikingly, induction of CXCL12 moderately promoted growth in LM3-transfected cells (Figure 2c). The results indicated that CXCL12 engagement to CXCR7 can induce mitogenic response, meanwhile, addition of CXCL12 may be essential to induce growth in cells with depleted CXCR7 but in the presence of CXCR4 (like LM3-shCXCR7-1 cells).

Next, we performed a cell cycle analysis to determine whether CXCR7 enhances cell growth via alteration of the cell cycle. As expected, CXCR7 overexpression triggered G_0_ quiescent cells in G_1_ phase and marked HepG2 and Hep3B cells progress through S+G_2_/M phases of cell cycle. Likewise, reduction of CXCR7 in LM3 and 97H altered the cell cycle by arresting the G_0_/G_1_ to S phase transition (Figure 2d, Supplementary Figure S1c). The results indicate a role for CXCR7 in HCC cells as a cell cycle priming factor.

### CXCR7 promotes tumorigenicity *in vivo*

**Figure 3:**
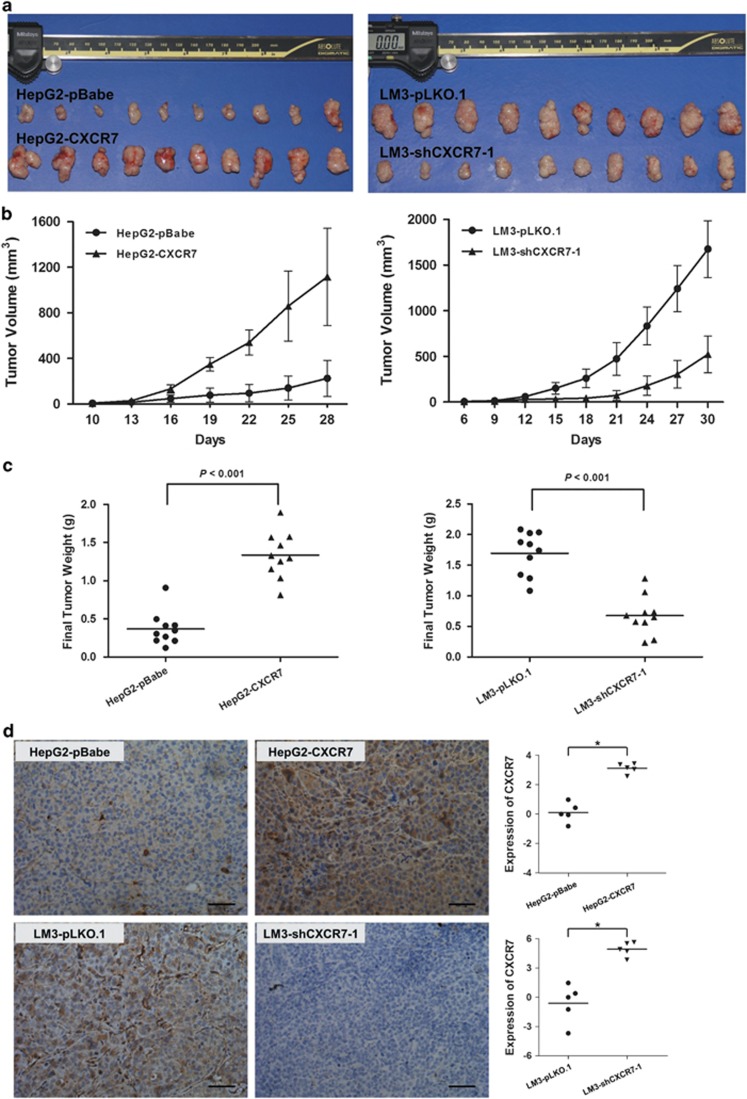
CXCR7 promotes *in vivo* tumorigenicity. (**a**) Macroscopic images were shown as isolated tumors. (**b**, **c**) CXCR7 promoted tumor growth in both size and weight via subcutaneous injection of established stable cells, while cells carrying empty vector (pBabe or pLKO.1) were used as controls (*P<0.05). (**d**) Five tumor implants were analyzed per condition by immunostaining for CXCR7. Quantitative evaluation was analyzed and presented in the right panel (*P<0.05)

To investigate whether CXCR7 accelerates tumorigenesis *in vivo*, nude mice were implanted subcutaneously with the indicated stable cell lines; each overexpression or depletion CXCR7 group contained ten nude mice. Tumor growths were observed and the macroscopic images were shown as isolated tumors, which presented that increased exogenous CXCR7 expression in HepG2 cells generated larger tumors than control cells, whereas silencing CXCR7 expression in LM3 cells formed smaller tumors (Figure 3a). In addition, tumor diameters were measured every 3 days with an electronic digital caliper, and it could be demonstrated clearly that HepG2 cells with transfected pBabe-CXCR7 showed promotion in both size and weight in tumor growth, while LM3 cells with transfected pLKO.1-shCXCR7-1 showed suppression of tumor growth, which statistically differed from those of their vector control (*P*<0.05, *t*-test; Figures 3b and c). Immunohistochemistry staining for CXCR7 expression in tumor tissues comfirmed the up or downtransfection efficiency in each group (Figure 3d). Therefore, CXCR7 can affect tumorigenesis substantially by favoring cell proliferation and the alteration of cell cycle features.

### CXCR7 promotes tumor angiogenesis and proliferation

**Figure 4:**
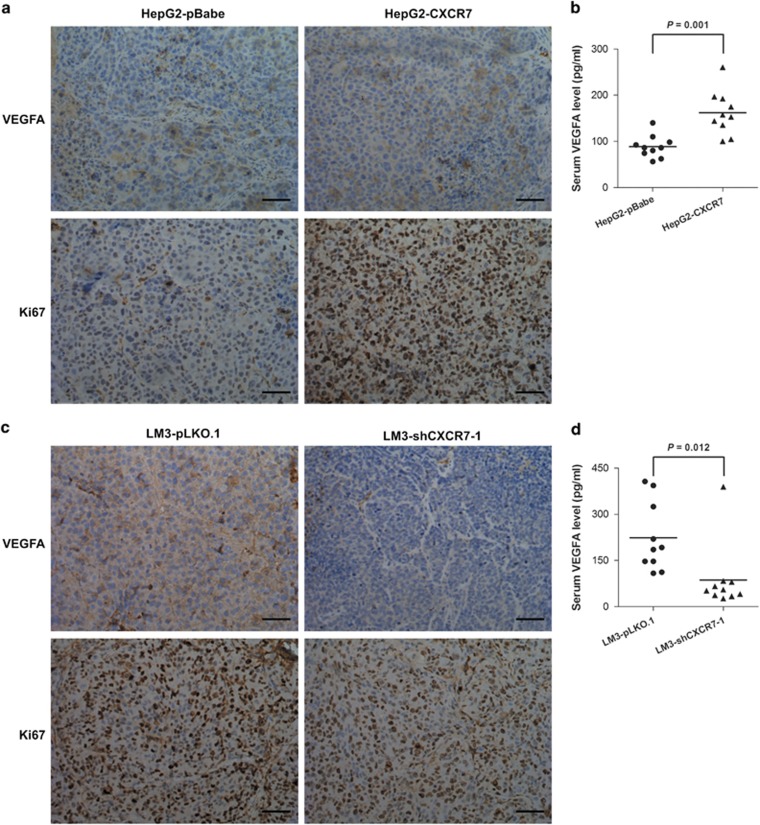
CXCR7 promotes tumor angiogenesis and cell proliferation. (**a**, **c**) Immnohistochemistry staining for VEGFA and Ki67 in tumor tissues from mice with subcutaneous HCC implantation. Five tumor implants were analyzed per condition (magnification × 200; scale bars, 100 *μ*m). (**b**, **d**) Regulation of serum VEGFA levels by CXCR7 expression were evaluated by ELISA

CXCR7 expression pattern during embryogenesis suggest a role in vasculogenesis and angiogenesis.^32^ Here, we investigated the proliferative and pro-angiogenic effects of CXCR7. The average levels of vascular endothelial growth factor A (VEGFA) in both the tumor tissues and serum of subcutaneous implantation models bearing CXCR7-transfectant HepG2 were significantly higher than that of control (*P*<0.05) (Figures 4a and b). Reverse effects were observed, the CXCR7 knockdown tumor tissues and serum did exhibit less vascularized behavior than their sh-controls (Figures 4c and d). Ki-67 staining demonstrated that the tumors derived from CXCR7-overexpressing cells were more proliferative than those derived from cells expressing vectors (Figure 4a). Tumor proliferation were suppressed in tumor tissues of the implantation models by knockdown of CXCR7 in LM3 cells (Figure 4c). Taken together, CXCR7 promotes tumor development by enhancing the expression and secretion of the proangiogenic factors VEGFA, which are likely to regulate tumor angiogenesis.

### CXCR7 mediates activation of MAPK signaling pathway

**Figure 5:**
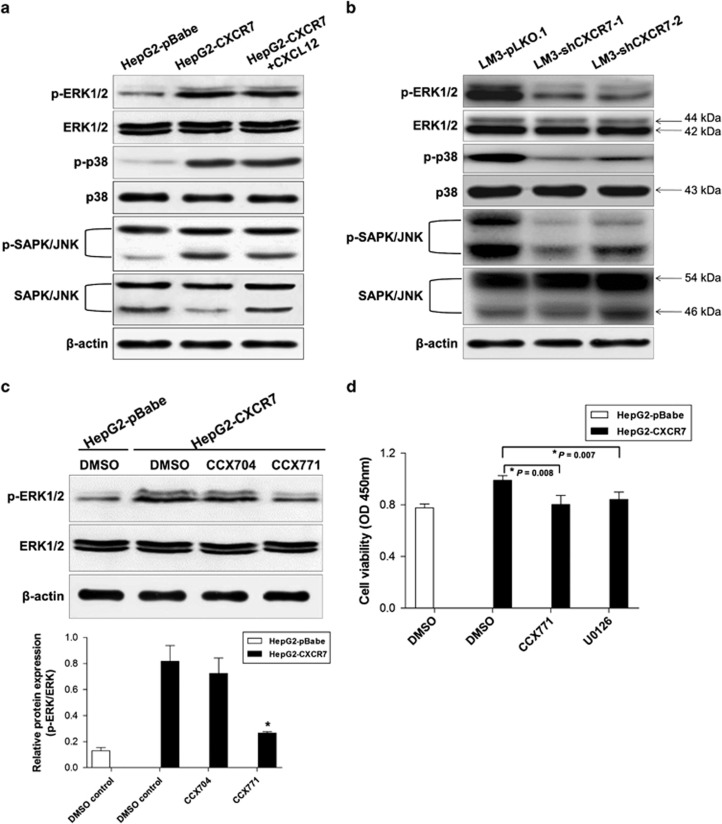
CXCR7 regulates activation of the MAPK signaling pathway. (**a**) Overexpression of CXCR7 led to enhanced phosphorylation of ERK1/2 and p38, but phosphorylation levels remained unchanged in HepG2 cells exposed to CXCL12 (100 ng/ml) for 30 min. Total ERK, p38, SAPK/JNK and *β*-actin were used as loading controls. (**b**) Knockdown of CXCR7 reduced intense phosphorylation of MAPK pathway proteins. (**c**) Expression profile of total ERK1/2 and phospho-ERK1/2 after blocking by CCX771, selective CXCR7 antagonists. Cell extracts were pretreated with DMSO or negative control CCX704 or CXCR7 inhibitor CCX771 for 24 h with 0.5 *μ*M concentration. Densitometry analysis by Quantity One shows quantitation of phospho-ERK1/2 levels, normalized against total ERK1/2. (**d**) Cells were seeded into 96-well plates (5 × 10^3^ cells/well) and cultured for 24  h in complete medium. Then HepG2-transfectant cells were cultured with DMSO, CCX771 (0.5 *μ*M), U0126 (25 mM) for 24 h. Cell proliferations were significantly decreased after treatment with CCX771 (CXCR7 inhibitor) or U0126 (ERK1/2 inhibitor). Each column represents mean±S.D. of three independent experiments. * denotes statistically significant difference (*P*<0.05) with respect to HepG2-CXCR7 cells

Although lacking classical GPCR characteristics, CXCR7 recruits *β*-arrestin-2 and signals efficiently through this protein.^33,34^ Recent studies indicate that CXCR7 may regulate other pathways including epidermal growth factor receptor (EGFR)/extracellular regulated kinase (ERK) axis,^30^ AKT pathway^35^ and mTOR signaling.^36^ To identify pathways which are involved in HCC signal transduction from CXCR7, several selected signaling molecules were examined. Overexpression of CXCR7 in HepG2 cells markedly enhanced phosphorylation of ERK1/2 and p38 but not SAPK/JNK. Strangely, phosphorylation levels remained unchanged in cultures exposed to recombinant CXCL12 (100 ng/ml) in HepG2 cells, indicating ligand-independent role of CXCR7 in mitogen-activated protein kinase (MAPK) activation (Figure 5a). Knockdown of CXCR7 in LM3 cells reduced intense phosphorylation levels of MAPK pathway proteins including ERK1/2 at Thr202/Tyr204, p38 at Thr180/Tyr182 and SAPK/JNK at Thr183/Tyr185 compared with its sh-control cells (Figure 5b).

Interestingly, by using CCX771, a selective CXCR7 antagonist, drastically inhibited CXCR7-induced phosphorylation of ERK1/2 signaling compared with negative control CCX704 in CXCR7-overexpressing HepG2 cells (Figure 5c). To assess the effects of ERK pathway on CXCR7-mediated cell proliferation, culture mediums containing CCX771 or U0126 at concentrations of 0.5 *μ*M and 25 *μ*M were added, respectively, to HepG2 cells stably expressing CXCR7. Culture medium containing DMSO alone was used as a control. Cell proliferations were markedly decreased in U0126 and CCX771 treatment groups (*P*<0.05) (Figure 5d), indicating that ERK1/2 is a functional target of CXCR7. Collectively, signaling by CXCR7 activates MAPK pathways. Furthermore, activation of CXCR7 increases the phosphorylation of ERK1/2 constitutively, and CXCR7-mediated proliferation is abrogated by ERK1/2 inhibition.

### CXCR7 enhances cell migration and invasion activity *in vitro*

**Figure 6:**
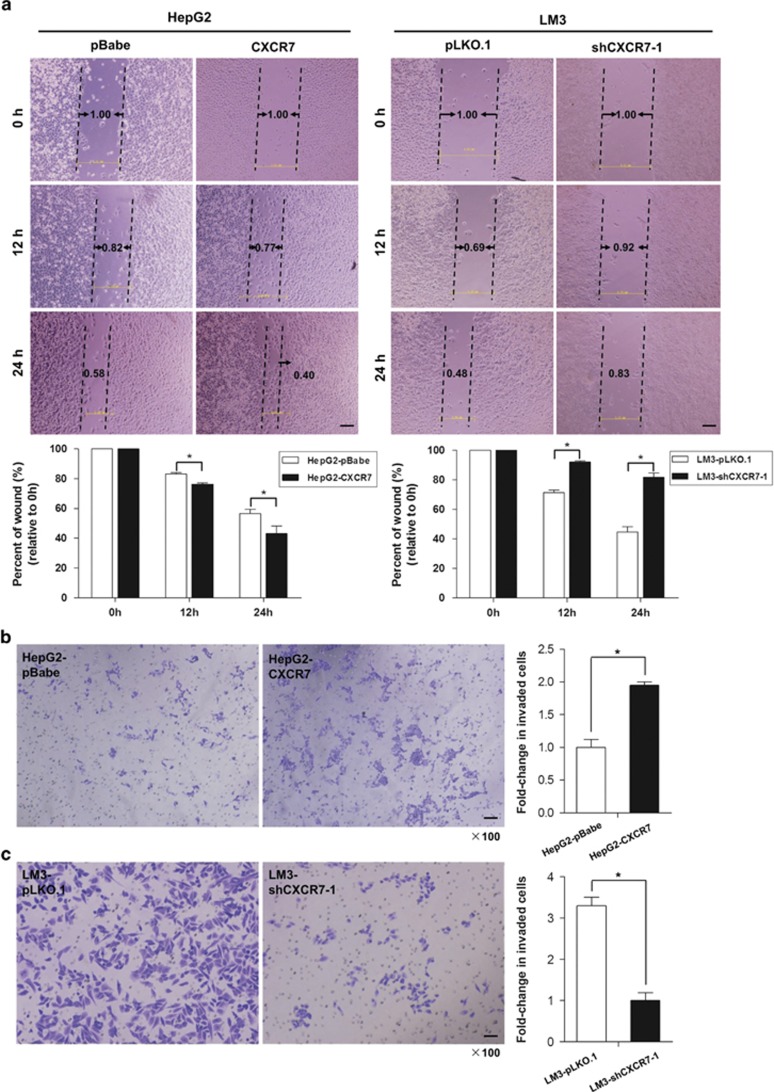
CXCR7 enhances cell migration and invasion *in vitro*. (**a**) In wound-healing experiment, the migration statuses of altered CXCR7 expression were assessed by measuring the movement of cells into a scraped area at indicated times. The wound width was measured and quantification analysis was presented in the lower panel. (**b**) Cell invasion after CXCR7 overexpression in HepG2 cells was measured using transwell assays. Values of mean±S.D. of triplicate experiments were plotted. (**c**) Cell invasive activity after CXCR7 knockdown in LM3 cells was measured and the summary graphs were presented (*P<0.05)

Besides enhanced growth advantage of tumor cell, increased cell invasion is a determinant hallmark of metastatic tumor cell. Thus we sought to explore the influence of CXCR7 on cell migration and invasion. Ectopic overexpression of CXCR7 in HepG2 cells significantly enhanced cell migration via wound-healing experiment; in contrast, the reversed effects were observed when CXCR7 was knocked down in LM3 cells (Figure 6a). Moreover, overexpression of CXCR7 promoted cell invasion (58 *versus* 112; *P*=0.045), while the number of invaded cells was significantly reduced in CXCR7 knockdown stable cells as compared with the sh-controls (181 *versus* 56; *P*=0.002) (Figures 6b and c), suggesting that increased CXCR7 expression promotes cell migration and invasion *in vitro*.

### CXCR7 increases lung metastasis *in vivo*

**Figure 7:**
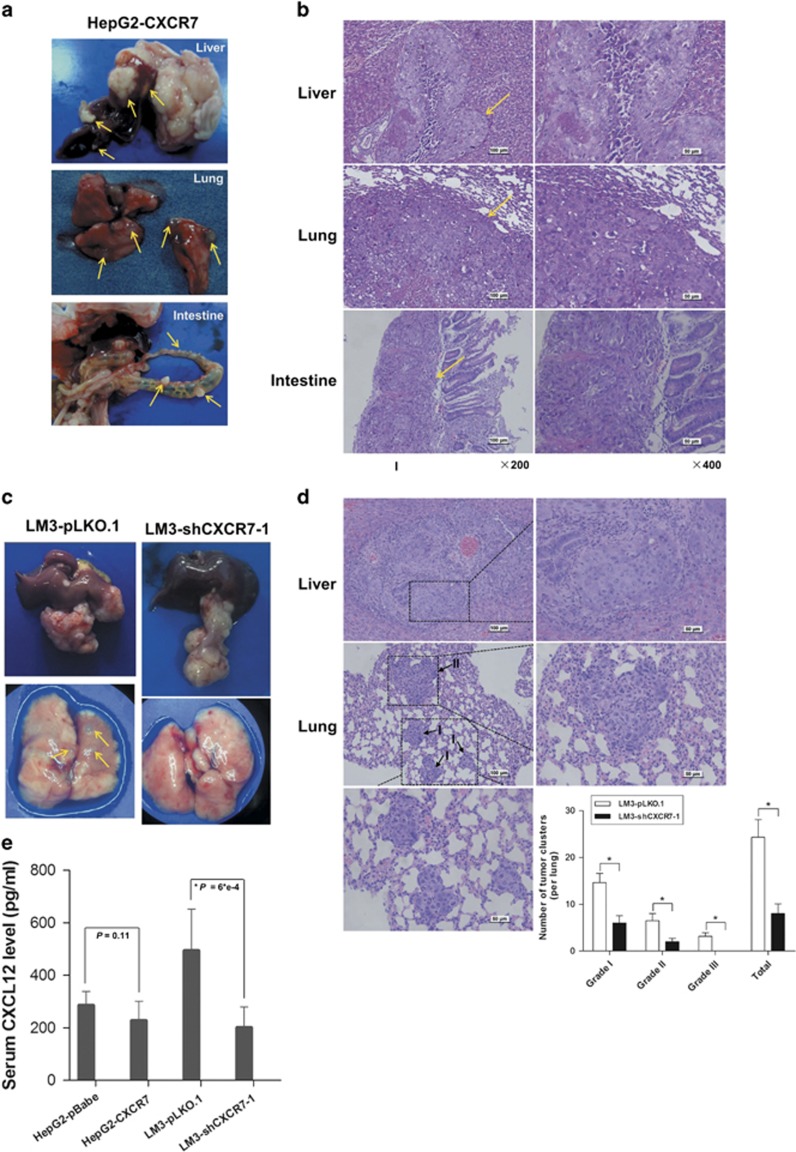
CXCR7 promotes *in vivo* tumor metastasis. (**a**) Macroscopic images in the orthotopic implantation models were shown as isolated liver, lung and intestine from CXCR7 overexpression group. Yellow arrows indicated metastatic nodules in each organ. No metastatic nodules were found in mice in control group. (**b**) Representative images of intrahepatic, pulmonary and intestinal metastatic foci (hematoxylin and eosin (H&E) stain) were shown in HepG2-CXCR7 group (Left panel: magnification × 200; scale bars 100 *μ*m; Right panel: magnification × 400; scale bars 50 *μ*m). (**c**, **d**) Reversed metastatic trends were observed when CXCR7 depletion tumor fragments were *in situ* liver inoculated into mice. Microphotographs of intrahepatic and pulmonary metastatic lesions were shown in LM3-pLKO.1 group by H&E staining. The total number and grades of lung metastatic lesions in the CXCR7-knockdown groups were much less than those in controls (*P<0.05). (**e**) CXCL12 levels in the serum of nude mice models with orthotopic implantation were detected by ELISA

To further determine the effects of CXCR7 on *in vivo* tumor metastasis, HepG2 or LM3 tumor xenografts were isolated from the foregoing subcutaneous tumor specimens and implanted into the liver to establish orthotopic models, and each overexpression or depletion CXCR7 group contained eight mice. In this study, implanted fragments survived and all mice formed tumor nodules at liver. The average volume of HepG2 orthotopic tumor in the CXCR7 overexpression group was noticeably bigger than the control group (data not shown). Lung metastases were visible in two (25%) mice of the CXCR7 group by hematoxylin and eosin (H&E) staining, while no lung metastases were found in the control group (Figures 7a and b). In consideration of CXCR7 knockdown situation, the volume of orthotopic tumor in shCXCR7 group was slightly smaller than the LM3-pLKO.1 group. In addition, the incidence of lung metastases of orthotopic tumor in LM3-shCXCR7-1 group and the sh-control group was 50% and 75%, respectively. The total number and grade of lung metastatic lesions in the shCXCR7 group was much lower than the sh-control (*P*<0.01) (Figures 7c and d,Supplementary Figure 3a). The average levels of CXCL12 in serum of the orthotopic models bearing CXCR7-transfectant HepG2 cells were slightly lower than that of the controls (228±73 pg/ml *versus* 286±52 pg/ml; NS), however, CXCL12 levels decreased by 2.5-fold in LM3-shCXCR7-1 groups (*P*<0.001) when compared with the sh-control groups (Figure 7e). Moreover, in LM3-pLKO.1 groups, we found lymphocytic infiltration of healthy parenchyma in liver and intestine, which were rarely detected in the CXCR7-knockdown groups (Supplementary Figure 3b). These results indicate that CXCR7 expression has a crucial role in metastasis of HCC, likewise, serum levels of CXCL12 are associated with inflammation.

### CXCR7 induces alterations of protein levels in cell supernatant

**Figure 8:**
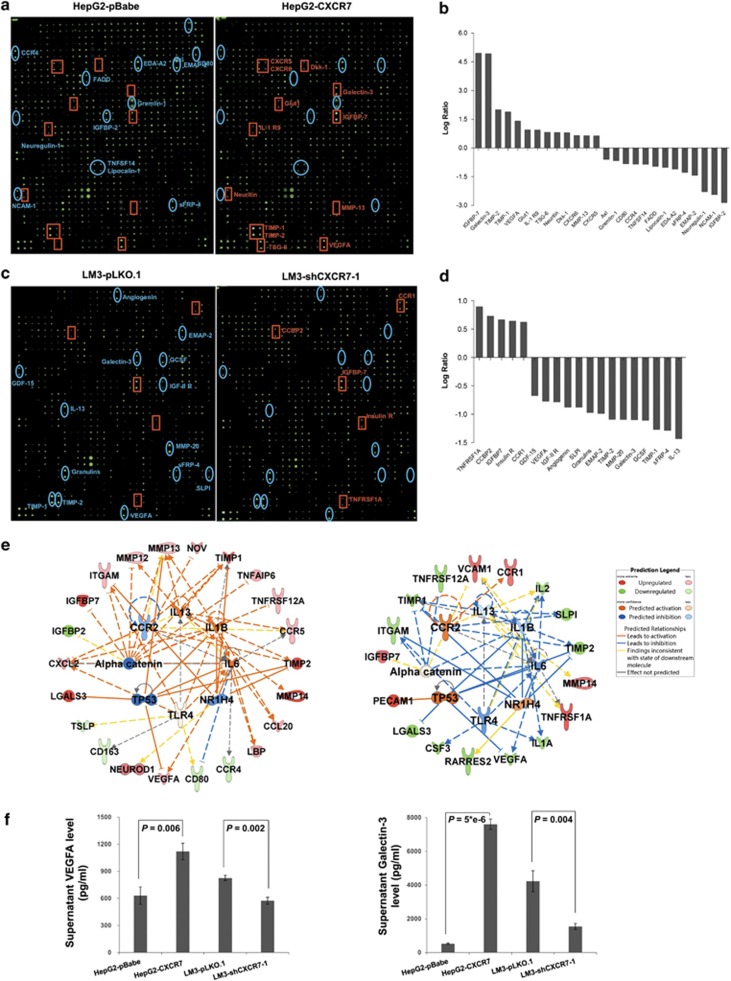
CXCR7 expression regulated secretion of VEGFA and galectin-3. The expression of proteins in culture media derived from HepG2-overexpessing (**a**) or LM3-reducing CXCR7 transfectants (**c**) were measured by RayBio antibody arrays. Parental cells with empty vector were used as controls. The images were visualized by fluorescence-conjugated streptavidin and captured using a laser scanner. (**b**, **d**) A standard 1.5-fold cutoff value for significance in protein abundance has been implemented to quantify up and downregulation of the antigens. Signal intensities are normalized using internal controls. The log-fold changes were graphically represented for the quantification of differential expressed proteins, that 26 and 19 differential molecules in CXCR7-overexpression and CXCR7-depletion subgroups respectively. (**e**) Comparative analyses of CXCR7 up and downregulation were run, and the statistically significant transcription factors were visualized in a network. Upstream analyses by IPA predict which transcription factors are activated or inhibited, based on the foregoing differentially expressed proteins. Gene products are represented as nodes. The red nodes are the upregulated proteins by altered CXCR7; the green nodes are the reduced ones. The inner ring theme colors of orange and blue, with orange indicating ‘activated' and blue reflecting ‘inhibited'. The inset image shows the prediction legend. (**f**) Supernatant levels of VEGFA and galectin-3 were evaluated via ELISA. The readouts were normalized by cell number. Error bars represent ±S.D. (*P*<0.05)

To explore potential cellular mediators induced or regulated by CXCR7 in HCC cells, we applied conditioned media (CM) onto biotin-label-based antibody arrays, which allow for simultaneous detection of 507 human proteins including many cytokines. Signal intensity ratio of >1.5 (log-fold change 0.58) or <0.67 (log-fold change −0.58) indicate significant differences in protein abundances. For CXCR7-overexpressing HepG2 cells, 26 proteins were differentially expressed including 13 increased and 13 decreased, compared with the controls (Figures 8a and b,Supplementary Table S2). Among them, the galectin-3 level increased 30-fold and VEGFA level increased twofold. The results were independently confirmed by ELISA (Figure 8f). For CXCR7-depleted LM3 cells, 19 differentially expressed proteins were identified including 5 upregulated and 14 downregulated proteins compared with its sh-controls. (Figures 8c and d,Supplementary Table S3). Among them, IL-13 exhibited the strongest discrimination power with a log-fold change of −1.4. Supernatant levels of VEGFA and Galectin-3 were decreased and confirmed by ELISA (Figure 8f).

Upstream analyses of CXCR7 up and downregulation were run by IPA, the most statistically significant transcription factors were quickly prioritized and then visualized in networks. Regulators including TP53, Alpha catenin, CCR2 and NR1H4 predicted inhibition while IL6, IL1B, IL-13 and TLR4 predicted activation in CXCR7-overexpression group (Figure 8e); while the reversed trends were observed in CXCR7-depletion subgroup in the right panel of Figure 8e. Upregulated levels of TIMP-2, LGALS3 (Galectin-3), VEGFA and MMP-13 predicted TP53 inhibition, which led to activate signal transduction. Reversely, downregulated levels of LGALS3 (Galectin-3) and VEGFA in LM3-shCXCR7-1 CM compared with its sh-control indicated TP53 activation, which caused the inactivation of signal transduction. IPA analysis also showed the pro-inflammatory pathways involved in CXCR7 overexpression group with IL13, IL6, IL1B and TLR4 activated, whereas anti-inflammatory pathways in CXCR7 depletion group. Moreover, interactive networks generated by IPA integrated the information of the differentially expressed proteins (Supplementary Figure S4). Collectively, proteomic approaches reveal that a large number of CXCR7-regulated proteins are involved in cell-matrix interactions, cell proliferation, cell survival and angiogenesis. Moreover, the potential downstream targets of CXCR7 are VEGFA and galectin-3, which are likely to participate in the regulation of tumor angiogenesis and contribute to the invasiveness of HCC cells.

## Discussion

Augmenting evidence accumulates that CXCL12 and its receptors, both CXCR4 and CXCR7, are involved in cancer development, affecting tumor cell adhesion, trans-endothelial migration, neovascularization and cell survival.^18,29,35^ In different tumor cell types, depending on differentiation status and environment, CXCR4 and CXCR7 may be expressed uniquely or in combination.^29,28^ As a result, studies on the cellular function of CXCR7 to date has produced a rather puzzling picture. Some groups provided evidence that CXCR7 represents a scavenger or a decoy chemokine receptor, which is responsible for either sequestering extracellular CXCL12^37,38^ or modulating CXCR4 signalling by forming CXCR7–CXCR4 heterodimers.^33,39^ In contrast, others using various tumor cells demonstrated that CXCR7 can actively control cell growth and survival, as well as cell adhesion and transendothelial migration.^18,25,26^ In our opinion, the biological significance of CXCR7, its signal transduction and further effects obviously depend on malignant cell types investigated.

It has been widely reported that CXCR7 expression is induced, compared with healthy tissues, in various types of cancer and is increased with malignancy. As for HCC, Monnier *et al.*^40^ reported that CXCR7 was differentially expressed in a cohort of 408 human HCC tissues, and that elevated expression of both CXCR7 and CXCL11 in tumor cells correlated with aggressive tumor behavior and poor prognosis. As depicted, we also found that CXCR7 expression was closely correlated to metastatic status in HCC cell lines and clinical samples. Through *in vitro* studies, we also found alterations in CXCR7 expression were positively correlated with the activities of proliferation, migration and invasion. In addition, upregulation of CXCR7 induced cell cycle progression while depletion of CXCR7 arrested the G_0_/G_1_ to S phase transition, which could partially explain the CXCR7-induced proliferating effects. Furthermore, CXCR7 stimulates ERK1/2 phosphorylation, but phospho-ERK1/2 levels remained unchanged when HepG2-transfectants were exposed to CXCL12, indicating ligand-independent role of CXCR7 in MAPK activation. And inhibition of ERK pathway by using U0126 drastically reduced cell growth, linking CXCR7-mediated cell proliferation to ERK activation. This is partially inconsistent with several studies, suggesting that CXCL12 binding to CXCR7 activates the AKT and ERK pathways.^26,28,41^ Current reports confirmed that binding of CXCL12 to CXCR7 stabilizes the association of CXCR7 with *β*-arrestin-2, which potentiates CXCL12-mediated downstream signaling.^33,42^ Moreover, CXCR7 constitutively internalized and recycled from intracellular pool to the cell membrane, a dynamic and periodic process of regulating the levels of extracellular CXCL12 and the expression of CXCR4.^22^ However, Singh *et al.*^30^ also demonstrated ligand-independent growth promotion by CXCR7 in prostate cancer, that growth-promoting activity of CXCR7 does not require its known ligands but interacts with EGFR and causes EGFR phosphorylation. We concluded from our *in vitro* data that the membrane expression of CXCR7 can cause signaling in autocrine type and eventually activate MAPK pathways in HCCs.

Another interesting finding of this study is that introduction of CXCR7 into HepG2 was both necessary and sufficient for tumor to grow aggressively; while reduction of CXCR7 inhibited subcutaneous tumor growth and metastatic nodules formation. CXCR7 influenced lymphocytes entry into the liver or intestine via orthotopic implantation models, and CXCR7 downregulation decreased CXCL12 levels in the bloodstream of mice, indicating that CXCL12 levels may be regulated by CXCR7 and have a pro-inflammatory role during HCC development. As reported before, CXCL12 expression is highly induced during inflamamation by recruiting immune cells to inflamed tissues, as well as in pro-angiogenic environments.^43,44^ Taken together, our data support the notion that CXCR7 is causally connected to HCC growth and metastasis regulation.

Cellular communications between malignant cells are changed dynamically via their secreted proteins to impact relevant biological processes. In an attempt to shed light on the complex mechanisms involved in CXCR7 gene expression and CXCR7 specific functions, antibody arrays were applied to identify the unique CXCR7-induced ‘molecule signature' in HCC development. These high-throughput arrays revealed for the first time that enhanced synthesis and secretion of cytokines like VEGFA and galectin-3 participate in the regulation of tumor angiogenesis and contribute to CXCR7-mediated metastatic phenotype. Supporting a pro-angiogenic role for CXCR7, its expression induced VEGF secretion, resulting in a positive feedback that further enhanced CXCR7 expression.^31^ In detail, VEGFA enormously promotes angiogenesis and tumor neovascularization in response to increasing delivery of oxygen and nutrients.^45^ VEGF also triggers proliferation using MAPK-dependent pathway and migration through PI3K/PKC-dependent pathway.^46^ Moreover, galectin-3, a member of the *β*-galactoside-binding mammalian lectins, binding to endothelial cells also stimulates angiogenesis.^47^ Cleavage of galectin-3 in tumors is highly indicative of matrix metalloproteinase activity and increased serum galectin-3 has been noted in a variety of cancers, especially when they are metastatic.^48,49^ Combined with comparative analysis in this work, several regulators including TP53, IL-6 and IL-1*β* are the potential upstream regulators of CXCR7-induced signaling. For instance, the tumor suppressor gene TP53 is predicted to cause inhibition in CXCR7-overexpressed HepG2 cells, consistent with the observation that CXCR7 enhanced cell proliferation in HCCs.

In summary, our results show that CXCR7 expression is enhanced in HCC cells and specimens correlated with metastatic abilities. CXCR7 activation especially promotes proliferation by phosphorylation of ERK1/2, invasion and angiogenesis by regulation of VEGFA and galectin-3. CXCR7 is a potential therapeutic target in HCC, and *in vitro* and *in vivo* studies with CXCR7 antagonists should be implemented in the near future.

## Materials and Methods

### Tissue microarray and specimens

Tissue microarray construction was completed in the Liver Cancer Institute of Zhongshan Hospital with clinical HCC specimens obtained from a cohort of 48 patients. Their clinicopathological characteristics are presented in Supplementary Table S1. All patients were diagnosed with primary HCC, and none had received any preoperative cancer treatment. Ethical approval from the Zhongshan Hospital Research Ethics Committee and patient written informed consent were obtained.

### HCC cell lines and cell culture

As research models for HCC, L02 and QSG7701 are nontransformed hepatic cell lines, QGY7703, HepG2 and Hep3B are non-metastatic hepatoma cell lines while MHCC97L, MHCC97H, HCCLM3 and HCCLM6 are originated from MHCC97, with similar genetic background and progressively metastatic abilities (MHCC97L<MHCC97H<HCCLM3<HCCLM6).^50^ These cell lines were cultured in DMEM or RPMI 1640 medium (Hyclone, Logan, UT, USA) containing 10% fetal bovine serum (Gibco BRL, Grand Island, NY, USA), 100 IU/ml penicillin G and 100 mg/ml streptomycin sulfate (Hyclone).

### Construction of stable expressing CXCR7 cell lines

To establish stable CXCR7-expressing cells, pBabe-CXCR7 retroviruses vectors containing the full-length CXCR7 sequence were conducted. The recombinant vectors were co-transfected into HEK293T cells with VSVG and Gag using Lipofectamine 2000 (Invitrogen, Carlsbad, CA, USA). Then the retrovirus particles were harvested and used to infect cells with 8 ug/ml polybrene (Sigma, St. Louis, MO, USA). Stable pools were selected with 2 *μ*g/ml puromycin (AMRESCO, Solon, OH, USA) for HepG2 and Hep3B for 7 days. Additional cloning protocols are provided in Supplementary Information.

### Stable silencing of CXCR7 expression by short hairpin RNA

Two pairs of shRNAs, named shCXCR7-1 and shCXCR7-2, were used to silence CXCR7 expression according to common protocol. The lentiviral expressing vectors were stably transfected into HCC cells. Stable transfectants were selected from transfected cultures following 2 weeks in puromycin selection medium (6.0 *μ*g/ml). Additional sequence information and protocols are provided in Supplementary Information.

### RNA isolation and real-time PCR

Total RNA was isolated from HCC cell lines using Trizol reagent (Invitrogen). Reverse transcription of cDNA synthesis was performed according to the manufacturer's protocols (Fermantas, Pittsburgh, PA, USA) and proceeded to real-time PCR on the 7500 Real-Time PCR (Applied Biosystems, Grand Island, NY, USA). Additional primer sequence information is provided in Supplementary Information.

### Western blots

Total cell lysates were prepared by RIPA buffer containing protease inhibitor (Roche, Basel, Switzerland). Protein concentrations were determined by BCA assay (Pierce, Rockford, IL, USA). Thirty micrograms of proteins were separated on SDS-PAGE and transferred to PVDF membranes (Millipore, Billerica, MA, USA). Membranes were then blocked in 5% fat-free milk and immunoblotted with appropriate antibodies. Detailed descriptions of the materials and methods can be found in the Supplementary Information.

### Flow cytometry analysis

The surface expression of CXCR7 on established stable cell lines were evaluated by FACS analysis, using standard procedures. The CXCR7 antigen was detected with a phycoerythrin–anti-CXCR7 monoclonal antibody (BioLegend, 8F11-M16, San Diego, CA, USA) or an IgG isotype-matched control (BioLegend, MOPC-173). Ten thousand cells from each sample were evaluated for fluorescence detection and the data were analyzed with CellQuest software (BD Biosciences, San Jose, CA, USA).

### Proliferation assay

Cell proliferation was evaluated over a 7-day period by the Cell Counting Kit-8 (Dojindo, Tokyo, Japan). After a 24 h serum withdrawal, 2 × 10^4^ cells were plated into 96-well flat-bottomed plates in 0.1 ml growth medium with or without CXCL12 (100 ng/ml). CCK-8 solution was added 10 *μ*l/well, cells were subsequently incubated for 2 h at 37 °C, and the data were read on a multiwell scanning spectrophotometer (Thermo Scientific, Waltham, MA, USA) at A_450_ and A_630_ for wavelength correction.

### Cell migration and invasion

Wound healing assay was used to evaluate cell migration ability. Serum was withdrawn before analysis to avoid effect of cell proliferation. The migration status was assessed by measuring the movement of cells into the scratched area created by a 10 *μ*l pipette tube, and the spread of wound closure was observed at indicated times, then photographed under a × 10 objective lens.

Cell invasive ability was determined using transwell chambers coated with a extracellular matrix gel (BD Biosciences). 5 × 10^4^ cells/well were placed in the pre-coated upper chamber and cultured with serum-free DMEM. The lower chamber contained 300 *μ*l complete media. After 48 h incubation, the cells on the upper surface of the filter were removed. Then the filters were fixed with 4% paraformaldehyde for 15 min and stained with crystal violet stain for 30 min (Sigma). The invasive activity was quantified by counting the number of trespassed cells per five high-power fields (magnification, × 100) from three independent experiments (mean±S.D.), and a *t*-test was used to show significant differences between two groups (*P*<0.05).

### Cell cycle analysis

To determine cell cycle profile, 2 × 10^6^ cells were harvested and fixed in cold 70% ethanol at −20 °C overnight. Then the cells were washed in PBS and the supernatant was discarded. cells were treated with 100 *μ*l of 100 *μ*g/ml RNase ribonuclease I at 37 °C for 30 min. PI (light sensitive; 500 *μ*l of 50 *μ*g/ml) was added and incubated at room temperature for 10 min, the samples were placed in Falcon tubes and analyzed by FACScan, and the percentages of cells occupying in the G_0_/G_1_, S, and G_2_/M phases were calculated from the resulting DNA histogram with CellQuest software (BD Biosciences).

### *In vivo* assays for tumor growth and metastasis

All experimental animal procedures were approved by the Animal Care and Use Committee of Fudan University. Five to seven-weeks-old male athymic nude mice (Shanghai Laboratory Animal Center, CAS) were maintained in individually ventilated caging systems in groups of five at 19–23 °C, with a 12-h light-dark cycle. Established stable cells (~5 × 10^6^) were injected subcutaneously into the right flank of each mouse. Mice were monitored daily, and tumor volumes were evaluated every 3 days. Tumor weights were measured after dissection at the end of the experiment.

Subcutaneous tumors were removed and dissected into 1-mm^3^ fragments, which were incubated into the liver of nude mice to establish orthotopic implantation models.^51^ Mice were killed 6 weeks later. At autopsy, tumors, livers, lungs, intestines and other organs were removed, fixed in formalin, and embedded in paraffin. Consecutive sections were made for every lung tissue block and stained with hematoxylin and eosin. Lung metastases were evaluated independently by two pathologists, and the number of metastatic lesions was calculated simultaneously. The formula of tumor volume and criteria of pathological staging in lung metastases can be found in Supplementary Information.

### Immunohistochemistry staining

Formalin-fixed, paraffin-embedded tissues were cut into 4-*μ*m sections. Following deparaffinization, sections were rehydrated and subjected to antigen retrieval. Sections were incubated at 4 °C overnight with target antibodies. Detailed descriptions of the materials and methods can be found in Supplementary Information.

### Cytokine antibody arrays

Soluble proteins in the culture medium of each established stable cell lines were measured using the Human Cytokine Array (RayBiotech, AAH-BLM-1-4, Norcross, GA, USA), according to the recommended protocols. Cells were plated 3 days before the experiment and were 80–90% confluent when the culture mediums were collected. Protein concentration of each sample was measured, then the culture supernate was dialyzed and biotinylated. The signals were imaged on the Odyssey infrared imaging system (LI-COR Biosciences, Lincoln, NE, USA) and then normalized by internal controls, and the values for cytokines in clear medium containing 10% FBS were background subtracted.

### ELISA assay

VEGFA and CXCL12 levels in mice serum were detected by ELISA (Boster Co., Wuhan, China), according to the recommended protocols. Antibody sandwich ELISAs were also used to evaluate VEGFA and galectin-3 levels in the conditioned medium of established stable cell lines (R&D System, Minneapolis, MN, USA). ELISA plates were detected using a microplate reader (Multiskan MK3, Thermo Scientific) at 450 nm within 30 min, with wavelength correction at 570 nm.

### Network analysis

To determine which pathways are significantly regulated by alteration of CXCR7 expression, lists of proteins detected by antibody array (Supplementary Tables S2 and S3) were imported into the ingenuity pathway analysis (http://www.ingenuity.com; Ingenuity Systems) and core analyses were performed. The networks were merged and the results are shown.

### Statistical analysis

All *in vitro* experiments were conducted in triplicate and carried out on three separate occasions. Numerical data were expressed as mean±S.D. Statistically significant differences between the means for the different groups were determined by two-tailed unpaired Student's *t*-test and defined as *P<0.05.

## Abbreviations

HCC: hepatocellular carcinoma
GPCR: G protein-coupled receptor
VEGF: vascular endothelial growth factor
EGFR: epidermal growth factor receptor
MAPK: mitogen-activated protein kinase
ERK: extracellular regulated kinase
