# Supplementary material for: CXCR7 stimulates MAPK signaling to regulate hepatocellular carcinoma progression
Source: Cell Death Dis. 2014 Oct 23;5(10):e1488–. doi: 10.1038/cddis.2014.392 (PMC4649507; doi:10.1038/cddis.2014.392)
Supplement: Supplementary Information [file cddis2014392x1.doc]

**Supporting information**

**Supplementary materials and methods**

**Construction stable expressing CXCR7 cell lines**

The full-length CXCR7 ORF (GenBank accession number NM_020311.2.) was amplified by PCR. The primers contained *Eco*RI and *Bam*HI restrict enzyme sites. The primers were as follows: sense5’-CGGGATCCATGTACCCATACGATG-3’ and anti-sense5’-CGGGATCCATGTACCCATACGATG-3’. After digestion with *Eco*RI and *Bam*HI, the PCR product was inserted into the multiple cloning site of the pBabe plasmid expression vector.

**Stable silencing of CXCR7 expression by short hairpin RNA**

CXCR7 shRNA (short hairpin RNA) constructs were cloned into pLKO.1 plasmid under the control of U6 promoter for stable expression (Sigma).Three pairs of annealed DNA oligonucleotides were inserted between *Age*I and *Eco*RI restriction sites according to common protocol. The most effective pair of sequence targeted to human CXCR7 is: sense5’-CCGGTCTTCGTCATCGGCATGATTGCTCGAGCAAT

CATGCCGATGACGAAGATTTTTG-3’ and anti-sense5’-AATTCAAAAATCTTCG

TCATCGGCATGATTGCTCGAGCAATCATGCCGATGACGAAGA-3’, which was also named shCXCR7-1. The second effective sequence of shRNA is: sense5’-CCGGGCCAGGGAACTTCTCGGACATCTCGAGATGTCCGAGAAGTTCCCTGGCTTTTTG-3’ and anti-sense5’-AATTCAAAAAGCCAGGGAACTTCTCG

GACATCTCGAGATGTCCGAGAAGTTCCCTGGC-3’, which was also named shCXCR7-2. These two pairs of shRNAs were used to knockdown CXCR7 in LM3 cells while shCXCR7-1 used to silence CXCR7 in 97H cells. The lentiviral vector pLKO.1 was used as negative control. The lentiviral expressing vectors were co-transfected into HEK293T cells with psPAX2 and pMD2.G. Supernatants were collected 36-48 h after transfection, filtered through a 0.4 µm filter, and used directly to infect HCC cells. Two rounds of infection 8 h apart were usually sufficient. Stable transfectants were selected from transfected cultures following 2 weeks in puromycin selection medium and evaluating the emergent cell colonies for CXCR7 knockdown by qRT-PCR and immunoblotting with rabbit anti-CXCR7 IgG (catalogue no. ab38089; Abcam).

**RNA isolation and real-time PCR**

Total RNA was extracted using Trizol (Invitrogen). 1 µg of total RNA was reversely transcribed into first strand cDNA and equal volume of cDNA was proceeded to real-time PCR. The primer sequences were as follows: sense5’-AGCACAGCCAGGAAGGCGAG-3’ and anti-sense 5’-TCATAGCCTGTGG

TCTTGGC-3’ for human CXCR7; sense5’-GCCTTATCCTGCCTGGTATTGTC-3’ and anti-sense 5’-GCGAAGAAAGCCAGGATGAGGAT-3’ for human CXCR4; sense5’-GTAACCCGTTGAACCCCATT-3’ and anti-sense5’-CCATCCAATCGGTA

GTAGCG-3’ for 18s; The relative abundance of mRNA was calculated by normalization to 18s mRNA.

**Western Blotting**

Total protein was extracted by lysing cells in RIPA buffer containing protease inhibitor. Protein samples were separated on 10% sodium dodecyl sulfate polyacrylamide gel electrophoresis (SDS-PAGE) and transferred to polyvinylidene fluoride (PVDF) membranes. After blocking with 5% non-fat milk in TBS-T for 1 hour, membranes were incubated with the primary antibody. The following antibodies were used: anti-CXCR7 (1:250, Abcam), anti-CXCR4 (1:500, Abcam), anti- Phospho-ERK1/2 (Thr202/Tyr204) (1:1000), anti-Phospho-p38 (Thr180/Tyr182) (1:1000), anti-Phospho-SAPK/JNK (Thr183/Tyr185) (1:1000, Cell Signaling Technology). Total ERK, p38, SAPK/JNK (1:1000, Cell Signaling Technology) and β-actin (1:5000, Genescript) were used as loading controls. Goat-anti-rabbit IgG conjugated to horseradish peroxidase (HRP)(1:5000, Cwbiotech) was used as the secondary antibody. Proteins were detected using ECL detection system (Pierce).

***In vivo* assays for tumor growth and metastasis.**

All experiments were approved by The Animal Care and Use Committee of Fudan University. Animals were maintained in individually ventilated caging systems in groups of five at 19°C to 23°C, with a 12-hour light-dark cycle, and fed with a conventional diet. To investigate whether CXCR7 accelerates tumorigenesis in vivo, nude mice were implanted subcutaneously with the indicated stable cell lines. The animals were monitored daily, and tumor volumes were evaluated every 3 days starting at day 10 or day 6. Calculating the volume of tumor using the formula V=(ab^2^)/2 in mm^3^, where a and b are the longest and the shortest perpendicular diameters of the tumor, respectively. Tumor weights were taken at termination on day 28 or day 30 and prepared for histology.

A metastatic model of HCC was obtained via orthotopic implantation of a tumor fragment selected from the foregoing subcutaneous tumor specimens. Tumor pieces (1-mm^3^ in size) were transplanted into the liver of nude mice under specific-pathogen-free conditions. Mice were sacrificed 6 weeks later and metastases were considered to have occurred if at least one microscopic metastatic lesion was found in any organ. At autopsy, the livers, lymph nodes, lungs, intestines and other organs were resected and processed for routine gross and microscopic examination for metastasis assay. Lung metastases were examined independently by two pathologists, and the number of metastatic lesions was calculated simultaneously. Based on the number of HCC cells in the maximal section of the metastatic lesion, lung metastases were classified into four grades. Grade I was defined as having 20 tumor cells; grade II had 20 to 50 tumor cells; grade III had 50 to 100 tumor cells; grade IV had>100 tumor cells.

**Immunohistochemistry Staining**

Formalin-fixed, paraffin-embedded tissues were cut into 4-μm sections. Following deparaffinization, sections were rehydrated and subjected to antigen retrieval by microwaving in 10mM sodium citrate (pH 6) for 10 minutes. Sections were incubated at 4°C overnight with antibodies against CXCR7 (1:100, Abcam), VEGFA (1:100, Abcam) and Ki67 (1:300, Santa Cruz). Immunostaining was performed using EnVision™ Detection Kit, Peroxidase/DAB, Rabbit/Mouse (DakoCytomation) according to the manufacturer’s instructions. Protein staining was evaluated under a light microscope at 200× magnification (Leca). Staining intensity was analyzed by Image-Pro Plus 6.0 and presented in a graphic format using error bars with 95% confidence intervals (CI) staining intensity.
