# Supplementary material for: CXCR7 stimulates MAPK signaling to regulate hepatocellular carcinoma progression
Source: Cell Death Dis. 2014 Oct 23;5(10):e1488–. doi: 10.1038/cddis.2014.392 (PMC4649507; doi:10.1038/cddis.2014.392)
Supplement: Supplementary Figure Legends [file cddis2014392x2.doc]

**Supplementary Figure Legends**

**Supplementary Figure 1.** **CXCR7 promotes cell growth and induces G1 to S phase progression.** (**a**) FACS analysis of CXCR7 expression in Hep3B- and 97H- transfected cells. CXCR7 protein expression were evaluated by FACS analysis using PE-anti-CXCR7 antibody, and PE mouse IgG served as isotype control. (**b**) Cell proliferations were examined by CCK-8 experiments. Over-expression of CXCR7 in Hep3B cells led to accelerate cell proliferation, while stably knockdown CXCR7 in 97H cells suppressed cell growth. Western blot analysis in the insets indicated the expressed CXCR7 in these cell lines. Error bars represent +/- SD. A t test was used to show significant differences between 2 groups (*P*< 0.05). (**c**) Cell cycle profile was analyzed by FACS. Up-regulation of CXCR7 in Hep3B induced cell cycle progression from G_0_/G_1_ to S+G2/M phase, while depletion of CXCR7 in 97H altered the cell cycle by arresting the G_0_/G_1_ to S phase transition. The summary graphs are represent the mean ± S.D. of three independent experiments. * denotes significant difference from controls (*P*< 0.05).

**Supplementary Figure 2. Proliferation of LM3 cell in response to altered CXCR7 expression.** (**a**) FACS analysis of CXCR7 expression in transfected LM3 cell lines. CXCR7 was transiently overexpressed using a pcDNA3.1 vector (labeled as LM3-CXCR7/LM3 OE Control). Inhibition of CXCR7 was achieved using siRNA to target expression of the receptor. The cells were denoted as LM3-siCXCR7/LM3-siControl. CXCR7 protein expression were detected using PE-anti-CXCR7 antibody, and PE mouse IgG served as isotype control. (**b**) Cell growth were analyzed for up- and down-regulation of CXCR7 in LM3 transfected cell lines. Over-expression of CXCR7 increased the proliferation rate, whereas reducing the receptor expression decreased the effects. The experiments were repeated at least 3 times, and the symbols represent mean ± SD (* *P*< 0.05).

**Supplementary Figure 3. Knockdown of CXCR7 in LM3 cells inhibits HCC metastasis and reduces inflammation *in vivo*.** (**a**) Metastasis rates were examined via orthotopic liver implantation of LM3 stable clones. The number of lung metastatic lesions (yellow arrows) in the shCXCR7 group was much lower than the sh-control groups. Smaller lung metastatic nodules (black dashed box) were detected, compared with those in shCXCR7-1 group. (**b**) Consecutive sections were made for every liver and intestine tissue block and stained with H&E. Intrahepatic (left panel) and intra-intestinal (right panel) inflammation were found, as the lymphocytic infiltration of healthy parenchyma in the target organs.

**Supplementary Figure 4.** **Networks developed by IPA integrate CXCR7-induced differentially expressed proteins.** (**a**) Pathway analysis was used to interpret the 26 differential expressed proteins in CXCR7-overexpression cell cultured media. (**b**) 19 differential expressed proteins were observed in CXCR7-depletion cell supernatant. Generated networks, which are ordered by a score meaning significance, are associated to functions according to both up- and down-regulated identifiers. IPA top score networks were presented, and VEGFA and galectin-3 involved sub-networks were highlighted respectively. Gene products are represented as nodes and direct biological relationships between two nodes as a line. The red nodes are the up-regulated proteins by altered CXCR7; the green nodes are the reduced ones.
