# Supplementary material for: CXCR7 stimulates MAPK signaling to regulate hepatocellular carcinoma progression
Source: Cell Death Dis. 2014 Oct 23;5(10):e1488–. doi: 10.1038/cddis.2014.392 (PMC4649507; doi:10.1038/cddis.2014.392)
Supplement: Supplementary Tables [file cddis2014392x3.doc]

| **Supplementary Table S1.** Clinicopathological characteristics of HCC Patients (n=48) | | |
| --- | --- | --- |
| **Characteristics** | **Case number** | **%** |
| Sex  Female  Male  Age (years)  ≤50  >50  HBsAg  Negative  Positive  Cirrhosis  No  Yes  ALT (U/L)  ≤75  >75  AFP (ng/mL)  ≤20  >20  Tumor size (cm)  ≤5  >5  Tumor number  Single  Multiple  Tumor capsule  None  Complete  Tumor thrombus  No  Yes  Tumor differentiation  I-II  >II | 4  44  20  28  5  43  13  35  38  10  15  33  23  25  36  12  29  19  35  13  31  17 | 8.33  91.67  41.67  58.33  10.42  89.58  27.08  72.92  79.17  20.83  31.25  68.75  47.91  52.08  75.0  25.0  60.42  39.58  72.92  27.08  64.58  35.42 |

**Supplementary Table S2.** Detection of altered molecules secreted from HepG2 cells by overexpression of CXCR7.

|  | Gene Symbol | Definition | Mass (kDa) | Accession No. | HepG2-CXCR7  /HepG2-pBabe fold change |
| --- | --- | --- | --- | --- | --- |
| Up- | IGFBP7 | Insulin-like growth factor-binding protein 7 | 29.13 | Q16270 | 30.649 |
| regulated | LGALS3 | Galectin-3 | 26.152 | P17931 | 30.296 |
| proteins | TIMP2 | Metalloproteinase inhibitor 2 | 24.399 | P16035 | 3.966 |
|  | TIMP1 | Metalloproteinase inhibitor 1 | 23.171 | P01033 | 3.683 |
|  | VEGFA | Vascular endothelial growth factor A | 27.042 | P15692 | 2.017 |
|  | SLC2A1 | Solute carrier family 2, Glucose transporter type 1 | 54.084 | P11166 | 1.923 |
|  | IL1RAPL2 | X-linked interleukin-1 receptor accessory protein-like 2, IL-1 R9 | 78.67 | Q9NP60 | 1.913 |
|  | TNFAIP6 | Tumor necrosis factor-inducible gene 6 protein, TSG-6 | 31.203 | P98066 | 1.757 |
|  | NRN1 | Neuritin | 15.333 | Q9NPD7 | 1.746 |
|  | DKK1 | Dickkopf-related protein 1 | 28.672 | O94907 | 1.728 |
|  | CXCR6 | C-X-C chemokine receptor type 6 | 39.28 | O00574 | 1.565 |
|  | MMP13 | Collagenase 3 | 53.82 | P45452 | 1.551 |
|  | CXCR5 | C-X-C chemokine receptor type 5 | 41.955 | P32302 | 1.546 |
| Down- | AXL | Tyrosine-protein kinase receptor UFO | 98.336 | P30530 | 0.665 |
| regulated | GREM1 | Gremlin-1 | 20.697 | O60565 | 0.633 |
| proteins | CD80 | T-lymphocyte activation antigen CD80 | 33.048 | P33681 | 0.566 |
|  | CCR4 | C-C chemokine receptor type 4 | 41.403 | P51679 | 0.56 |
|  | TNFSF14 | Tumor necrosis factor ligand superfamily member 14 | 26.35 | O43557 | 0.558 |
|  | FADD | Protein FADD | 23.279 | Q13158 | 0.517 |
|  | LCN1 | Lipocalin-1 | 19.25 | P31025 | 0.497 |
|  | EDA2R | Tumor necrosis factor receptor superfamily member 27 | 32.759 | Q9HAV5 | 0.471 |
|  | SFRP4 | Secreted frizzled-related protein 4 | 39.827 | Q6FHJ7 | 0.415 |
|  | EML2 | Echinoderm microtubule-associated protein-like 2 | 70.679 | O95834 | 0.374 |
|  | NRG1 | Pro-neuregulin-1, membrane-bound isoform | 70.392 | Q02297 | 0.207 |
|  | NCAM1 | Neural cell adhesion molecule 1 | 94.574 | P13591 | 0.186 |
|  | IGFBP2 | Insulin-like growth factor-binding protein 2 | 34.814 | P18065 | 0.139 |

Notes: All quantitative data were normalized by internal controls according to the manufacture’s instructions and a standard 1.5-fold cutoff value was used to determine differentially expressed proteins.

**Supplementary Table S3. Detection of altered molecules secreted from LM3 cells by depletion of CXCR7.**

|  | Gene Symbol | Definition | Mass (kDa) | Accession No. | LM3-shCXCR7  /LM3-pLKO.1 fold change |
| --- | --- | --- | --- | --- | --- |
| Up- | TNFRSF1A | Tumor necrosis factor receptor superfamily member 1A | 50.495 | P19438 | 1.853 |
| regulated | CCBP2 | Chemokine-binding protein 2, Atypical chemokine receptor 2 | 43.443 | O00590 | 1.655 |
| proteins | IGFBP7 | Insulin-like growth factor-binding protein 7 | 29.13 | Q16270 | 1.581 |
|  | INSR | Insulin receptor | 156.333 | P06213 | 1.555 |
|  | CCR1 | C-C chemokine receptor type 1 | 41.173 | P32246 | 1.537 |
| Down- | GDF15 | Growth/differentiation factor 15 | 34.14 | Q99988 | 0.629 |
| regulated | VEGFA | Vascular endothelial growth factor A | 27.042 | P15692 | 0.587 |
| proteins | IGF2R | Cation-independent mannose-6-phosphate receptor | 274.375 | P11717 | 0.582 |
|  | ANG | Angiogenin | 16.55 | P03950 | 0.546 |
|  | SLPI | Antileukoproteinase | 14.326 | P03973 | 0.546 |
|  | GRN | Granulins | 63.544 | P28799 | 0.511 |
|  | EML2 | Echinoderm microtubule-associated protein-like 2 | 70.679 | O95834 | 0.506 |
|  | TIMP2 | Metalloproteinase inhibitor 2 | 24.399 | P16035 | 0.472 |
|  | MMP20 | Matrix metalloproteinase-20 | 54.387 | O60882 | 0.471 |
|  | LGALS3 | Galectin-3 | 26.152 | P17931 | 0.468 |
|  | CSF3 | Granulocyte colony-stimulating factor | 22.293 | P09919 | 0.466 |
|  | TIMP1 | Metalloproteinase inhibitor 1 | 23.171 | P01033 | 0.416 |
|  | SFRP4 | Secreted frizzled-related protein 4 | 39.827 | Q6FHJ7 | 0.411 |
|  | IL13 | Interleukin-13 | 15.816 | P35225 | 0.372 |

Notes: All quantitative data were normalized by internal controls according to the manufacture’s instructions and a standard 1.5-fold cutoff value was used to determine differentially expressed proteins.
